# Advancing fetal autopsy in cases of maceration: Underwater dissection technique and its forensic relevance

**DOI:** 10.1111/1556-4029.70336

**Published:** 2026-04-12

**Authors:** Francesca Buffelli, Sharon Duzioni, Valerio Gaetano Vellone, Alessandro Bonsignore, Ezio Fulcheri

**Affiliations:** ^1^ Pathology Unit IRCCS‐Istituto Giannina Gaslini Genoa Italy; ^2^ Department of Neurosciences, Rehabilitation, Ophthalmology, Genetics, Maternal and Child Health (DINOGMI) University of Genova Genoa Italy; ^3^ Section of Pathology, Department of Surgical Sciences and Integrated Diagnostics (DISC) University of Genova Genoa Italy; ^4^ Medico Legal Unit IRCCS‐Istituto Giannina Gaslini Genoa Italy; ^5^ Section of Legal and Forensic Medicine, Department of Health Sciences (DISSAL) University of Genova Genoa Italy

**Keywords:** autopsy technique, fetal autopsy, forensic pathology, intrauterine death, macerated fetuses, underwater dissection

## Abstract

Fetal autopsy remains essential for determining the cause of intrauterine death and for supporting clinical, genetic, and forensic evaluations. However, in cases of advanced maceration, autolysis severely compromises tissue integrity, often preventing adequate identification of anatomical structures and limiting the diagnostic and medico‐legal value of traditional autopsy. To address these challenges, we developed and applied an innovative underwater dissection technique designed to improve visualization, separation, and recovery of fragile organs in macerated fetuses. Seventeen cases of intrauterine fetal death between 16 and 36 weeks were examined using a standardized protocol performed entirely in a water‐filled basin. Hydrostatic buoyancy gently distended viscera, separated tissue planes, and reduced structural collapse, enabling controlled evisceration and minimizing tearing of friable organs. Thoraco‐abdominal dissection, removal of visceral blocks, and cranial extraction were performed under water, followed by fixation and histologic analysis. The technique consistently allowed recognition, retrieval, and documentation of all major organs, including those typically lost or unidentifiable in macerated conditions—such as the thymus, gonads, adrenal glands, and gastrointestinal tract. Improved visibility facilitated accurate photographic documentation and preserved organ relationships despite autolysis. Histology remained feasible in every case and yielded clinically and forensically relevant information regarding gestational age, congenital anomalies, infectious processes, and timing of intrauterine retention. This underwater dissection method is simple, reproducible, and it substantially enhances the diagnostic yield of fetal autopsy in macerated cases. By enabling better preservation and documentation of anatomical structures, it strengthens forensic interpretation and contributes to more reliable determinations of cause and timing of death.

**Highlights**

- Underwater dissection enables autopsy of fetuses in advanced maceration.
- Technique preserves anatomical integrity for forensic documentation.
- Improves recovery of delicate organs critical to forensic assessment.
- Supports genetic, microbiological, and legal investigations in perinatal death.
- Enhances diagnostic yield where conventional autopsy is inconclusive.

## 1 INTRODUCTION

In perinatal forensic medicine, autopsy of the macerated fetus is a key tool for clarifying the causes of intrauterine death [1, 2]. This is particularly relevant in cases of voluntary termination of pregnancy (VTP), selective abortion (also known as selective feticide), late spontaneous abortion (LSA), intrauterine fetal death (IUFD), malformative pathologies, or suspected maternal abuse of recreational or therapeutic substances. Such drug‐related conditions may mimic natural pathological or paraphysiological conditions. Beyond its clinical and scientific significance, this procedure is embedded within a defined regulatory framework that governs its purpose, methods, and medico‐legal implications.

Despite advances in prenatal diagnostics—especially high‐resolution ultrasound and fetal magnetic resonance imaging—autopsy remains the only definitive tool, particularly in complex cases involving multiple or syndromic malformations [3].

Autopsy analysis enables detailed phenotypic and histological characterization, distinguishing true malformations from developmental defects, epigenetic variants, and minor anomalies. It also facilitates the identification of dysplastic, dysmetabolic, or storage disorders and can be integrated with advanced cytological and molecular investigations.

### 1.1 Legal implications of autopsy

Fetal autopsy is an irreplaceable tool in the investigation of intrauterine death cases, not only for its diagnostic value but also for its significant medico‐legal implications. As noted by Cohen and Scheimberg [4], even in macerated fetuses—where autolysis compromises morphological integrity—autopsy still yields fundamental data for reconstructing the circumstances of death. By analyzing varied morphometrics, histological findings of organs (especially lungs, brain, and kidneys), and both morphological and microscopic placental features, it is possible to reliably estimate gestational age, establish the interval between death and delivery, and, most importantly, potentially distinguish stillbirth from live birth [5]. This distinction is critical in forensic medicine, particularly in cases of suspected obstetric negligence or maternal mistreatment.

Cassidy et al. [6], in a study of 385 fetal autopsies, showed that autopsy can confirm or revise prenatal diagnosis and, in selected cases (2.3%), significantly modify recurrence risk assessment. This finding is important not only for genetic counseling but also for the legal and insurance management of subsequent pregnancies, as it provides objective grounds for targeted clinical interventions or prevention strategies. The study further demonstrated that autopsy may reveal complex pathologies not identified prenatally, reinforcing its value in the forensic field as well.

The findings of Fatima et al. [7], although excluding macerated fetuses, also confirm the value of autopsy in determining causes of intrauterine death. Placental factors accounted for 43.6% of cases, followed by fetal causes (35.7%) and maternal causes (21.4%). Congenital anomalies, placental insufficiency, and infections (particularly chorioamnionitis) were the most frequent conditions identified.

The study also emphasizes the importance of placental examination and clinico‐pathological correlation. These are essential in the medico‐legal context for clarifying causal dynamics, establishing professional liability with greater certainty, and guiding potential litigation.

Therefore, even in cases of severe maceration, fetal autopsy not only retains its diagnostic value but also acquires significant probative weight in medico‐legal investigations. It contributes to protecting healthcare professionals, ensuring proper clinical and judicial management, and safeguarding both families and institutions.

## 2 PREFACE

Postmortem changes occurring in the absence of external microorganisms and scavenging animals or insects refer to the series of alterations that develop in the body after death. Understanding these processes is essential to avoid confusing them with pre‐existing pathological conditions, particularly in the fetus. Although cadaveric changes occur in all organisms, they present unique characteristics in fetuses [8].

Subsequent cadaveric phenomena include rigor mortis, cooling of the body, hypostasis, blood coagulation, and drying of the mucous membranes. Transformative phenomena include autolysis, cell damage caused by lytic enzymes within the cytoplasm—and autodigestion, the tissue breakdown due to digestive juices and enzymes released by specialized epithelia. In the fetus, putrefaction generally does not occur, since the uterine environment is sterile and closed (except in cases of severe ascending infections limited to the uterine cavity and fetoplacental unit) or when membranes rupture prematurely. In addition, the fetal gastrointestinal tract often lacks a fully colonized microbiome, which further contributes to the absence of putrefactive phenomena following intrauterine death. Instead, the fetal body undergoes maceration, followed by progressive dehydration leading to corification (“fetus papyraceus”) and calcification (“lithopedion” fetus).

Nevertheless, detailed evaluation of these changes can help clarify whether death was due to a natural event or to external factors such as violence or negligence in personal, familial, or obstetric care.

## 3 AUTOPSY PROCEDURE IN WATER

### 3.1 External examination

Before beginning the autopsy, a complete assessment of physical characteristics must be carried out, including fetal sex, development, trophism, and nutritional status.

It is equally important to evaluate the degree and extent of cadaveric changes, both secondary and transformative. In fetuses with obvious maceration, the autopsy may be performed either after fixation in formalin or directly after expulsion, following preservation in a refrigerated room at 5°C.

The body must be weighed and measured according to the autopsy protocol, bearing in mind that the values should be critically interpreted in light of the state of preservation. Measurements of circumferences (cranial, thoracic, abdominal) are largely irrelevant in these cases and should be reported as relative rather than absolute data. By contrast, the condition of the skin color, texture, and any degree of epidermal detachment must be described in detail. Crown‐rump and vertex‐calcaneal lengths must also be carefully measured, considering potential deformations caused by overlapping cranial bones (*Spalding phenomenon*) and the flattening of the calvaria.

Finally, a thorough evaluation should include the following:

- Choanae: assess patency and symmetry;
- Oral cavity: inspect the tongue, hard and soft palate;
- Umbilical cord: measure length and assess condition;
- Anal canal: check patency;
- Vaginal canal (female fetuses): check patency; and
- Testes (male fetuses): verify the level of descent of each testis.

### 3.2 Internal examination

For the autopsy, a glass tray measuring 60 × 50 cm and ~15 cm deep is filled with water.

**FIGURE 1:**
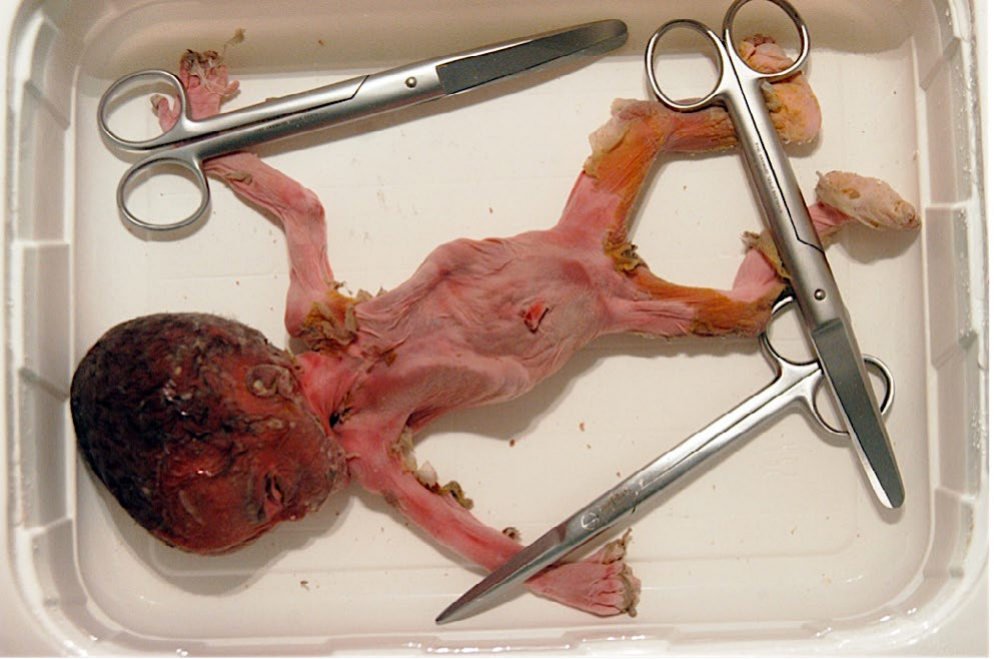
Fetus lying on the bottom of the tray and stabilized by the upper and lower limbs.

The fetus is placed on the bottom of the tray and stabilized with weights applied to the upper and lower limbs, preventing floating and ensuring they do not interfere with cutting or evisceration. After photographic documentation (Figure 1), the standard double Y‐shaped incision of the thoraco‐abdominal skin is performed (Figure S1). It begins bilaterally at the shoulders, converges at the mid‐sternum, continues down the midline to the suprapubic area, and bifurcates toward the inguinal regions.

The upper skin flap is flipped cranially over the neck, detaching the subcutaneous tissues and exposing the ribs, sternum manubrium, and sternocleidomastoid muscles. The lateral flaps are spread, and the insertions of the pectoralis major and diaphragm along the costal margin are cut, exposing the rib cage and part of the peritoneal cavity.

The skin flaps are secured with small Kocher clamps to prevent movement during the procedure.

During exposure of the abdominal region, the umbilical cord must be preserved. Once clamped, it allows the umbilical vein to be traced intrahepatically to its confluence with the ductus venosus (Aranzio's duct). The two arteries are examined along with the urachus and bladder dome. These three structures, continuous with the umbilicus, are finally reflected over the pubic symphysis.

#### 3.2.1 Evisceration

For the thoracic section, curved scissors are preferable to a scalpel, as they reduce the risk of injuring underlying organs.

The first step is to disarticulate the sternoclavicular joints bilaterally. Next, the ribs are cut on both sides with scissors, 3–4 mm lateral to the chondrosternal junction. The costosternal plate is then removed with forceps in the left hand, while the scalpel in the right hand is used to detach the anterior diaphragm insertions and fibrous adhesions connecting the plate to the mediastinum.

To visualize the ossification centers of the manubrium and sternal body—composed of cartilaginous sternebrae with central ossification nuclei indicating fetal development—a transillumination of the sternal plate is performed.

**FIGURE 2:**
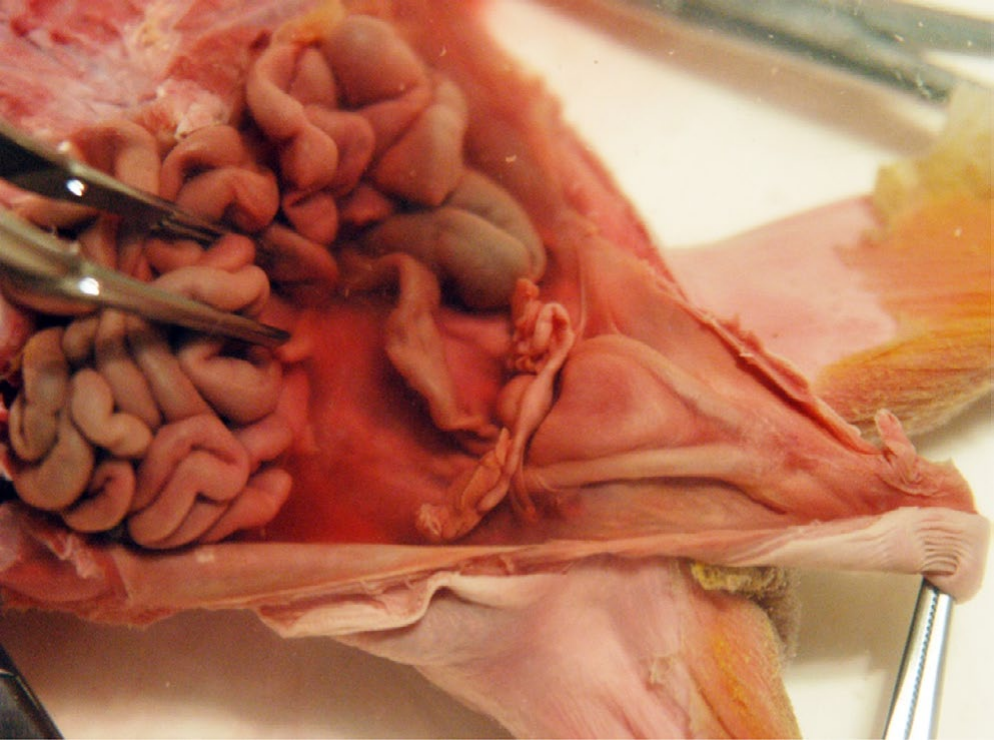
Exposure of the abdominal organs and female gonads.

After verifying the anatomical relationships of the thoracoabdominal organs, evisceration begins from below, at the pelvis, with removal of the gonads (Figure 2). This step is essential, as after bowel removal, the reference point is lost and the gonads—extremely delicate in macerated fetuses—are difficult to locate.

The large intestine is cut and clamped at the rectosigmoid junction, and the bowel is removed starting with the duodenum, dissecting along the mesenteric attachment. Scissors or a scalpel may be used. The cut must remain close to the intestinal wall to avoid torsion once the bowel is isolated.

**FIGURE 3:**
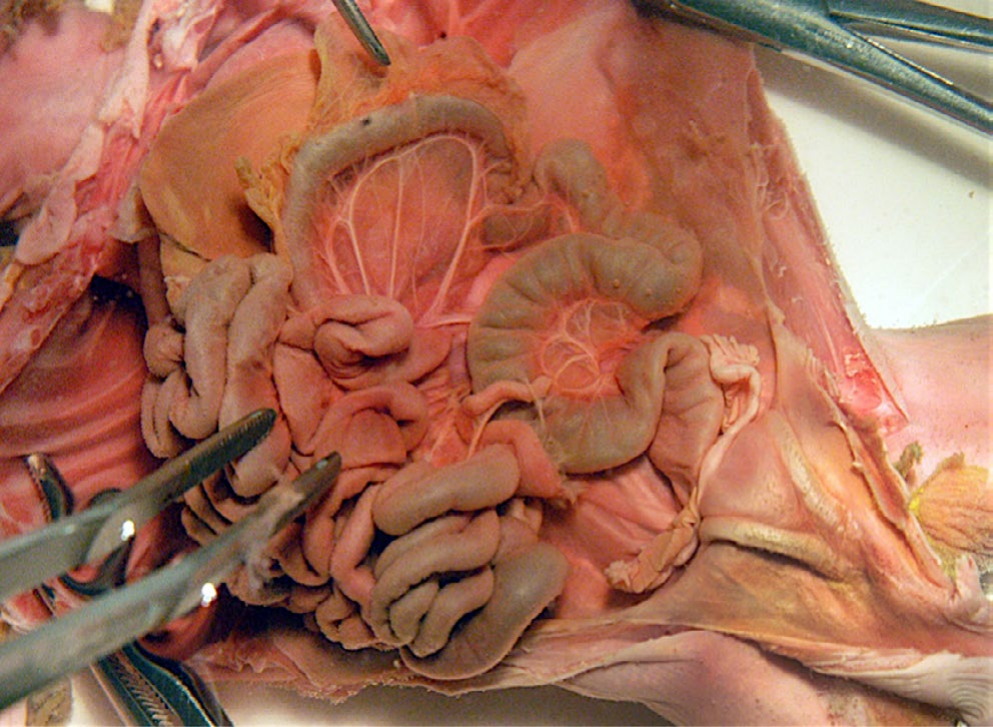
Exposure of the mesenteric fan, Riolano arches, and presence of meconium.

The small and large intestines are removed as a single block, with assessment of wall consistency and the presence of meconium (Figure 3).

Using the same “traction‐based removal technique” applied to the gonads, the spleen is excised after documenting its presence and location (to exclude situs inversus or isomerism).

The next block comprises the liver, stomach, duodenum, and pancreas, which are later separated for individual examination. Special attention is given to the liver, where patency of the ductus venosus (Aranzio's duct) must be checked to detect thrombotic occlusions. The umbilical vein pathway to the inferior vena cava is imagined, and slightly to the right of it, a deep 45° incision is made into the liver, exposing the umbilical vein, ductus venosus, suprahepatic veins, and inferior vena cava.

**FIGURE 4:**
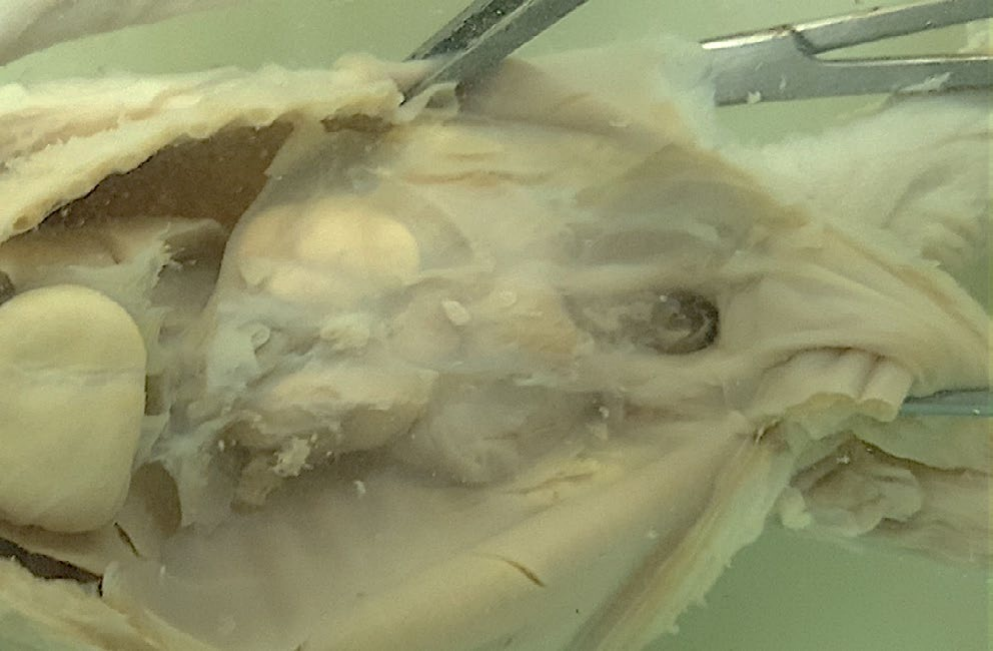
After removal of the abdominal organs, evidence of the kidneys in their correct anatomical position and the ureters running smoothly to the bladder.

The entire diaphragm is then removed. The Gerota fascia is incised to expose the retroperitoneal organs (Figure 4). The kidneys and adrenal glands are excised en bloc, tracing the ureters to the bladder. The pelvic organs are then removed en bloc as well.

For the thoracic cavity, scissors are used to separate the neck muscles from the deeper structures, exploring the two horns of the thymus. The thymus is lifted and detached from the parietal pericardium, great vessels, and brachiocephalic vein.

**FIGURE 5:**
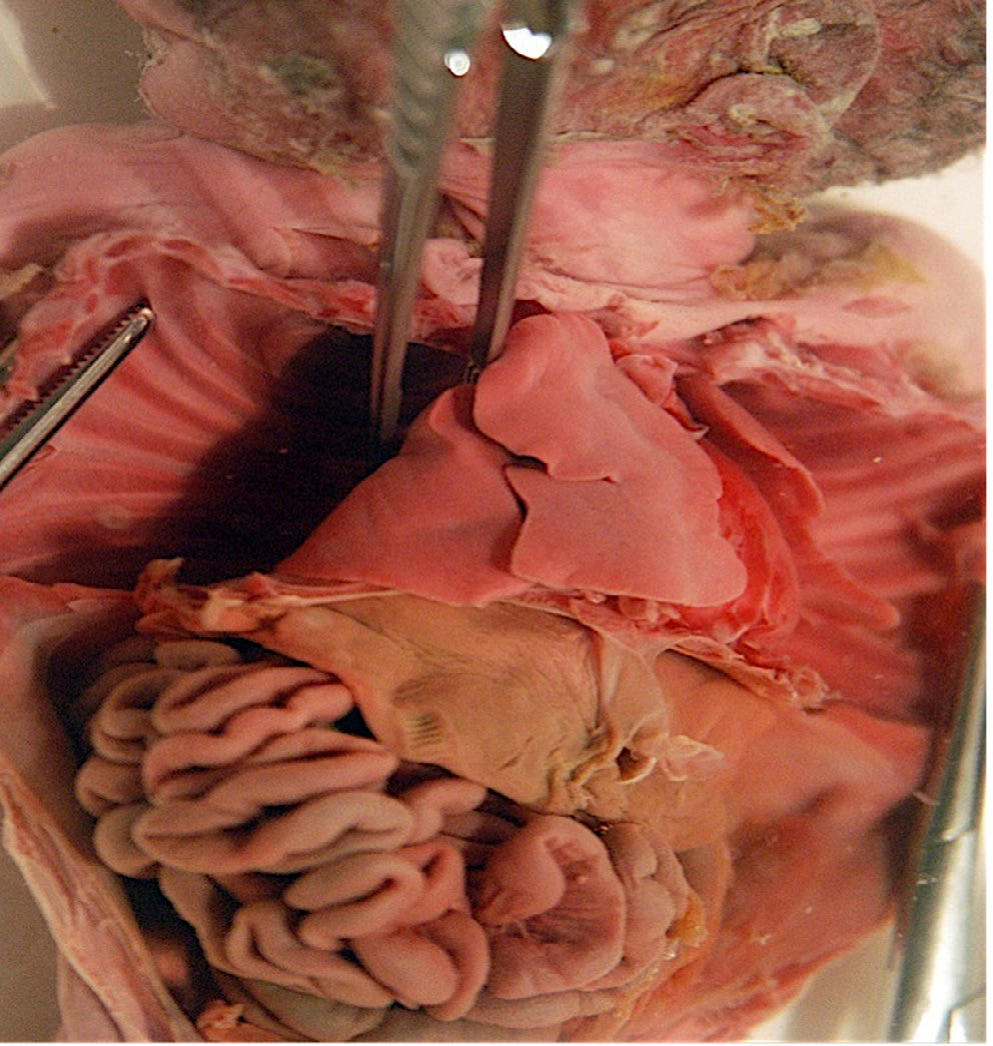
Exposure of the chest cavity with lungs in situ; the right lung appears trilobed.

**FIGURE 6:**
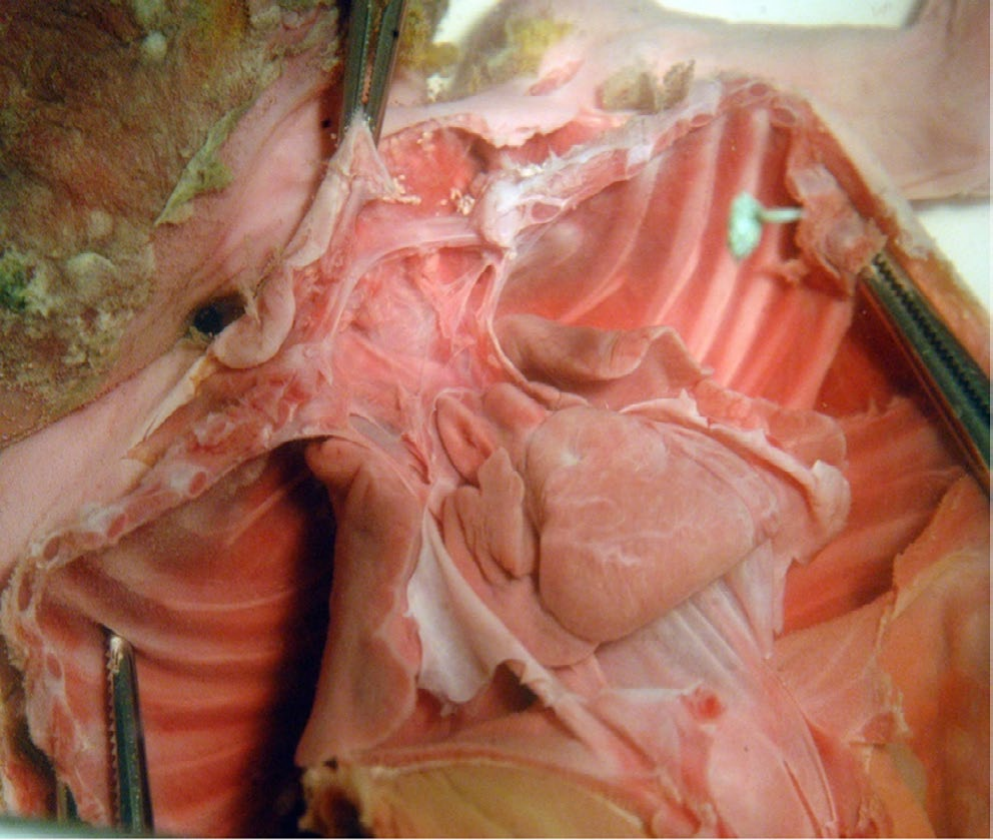
Heart in situ after removal of the pericardium; aortic arch and pulmonary arteries with regular course.

The heart‐lung block is then removed (Figure 5). This requires a transverse cut at the thyroid region, followed by dissection of the aorta along the vertebral column down to the iliac bifurcation. Before extraction, the pericardial sac is opened with an inverted Y incision, and the pericardium and loose connective tissues overlying the great vessels are removed (Figure 6).

Once the aorta and pulmonary artery are fully exposed, truncal pathologies can be ruled out. The connections between the heart, lungs, arteries, and veins are checked, and the lobation of each lung is documented. Finally, the lungs are separated from the heart and main bronchi.

#### 3.2.2 Opening of the skull

The goal of brain removal is to extract and fix the entire encephalon. In macerated fetuses, the brain is markedly softened, requiring extreme care during extraction.

A long scalp incision is made from one auricle to the other, passing over the occiput and the posterior fontanelle. The scalp is then reflected: the anterior flap is pulled forward over the frontal bones, while the posterior flap is folded back beneath the occipital bone. At this stage, the mobility of the cranial sutures and the contours of the fontanelles are evaluated.

Two sagittal incisions are then made along the lateral edges of the anterior fontanelle. From these points, parallel cuts are extended along both margins of the sagittal sinus to the lambdoid fontanelle. From there, incisions continue along the occipitoparietal sutures on both sides, ending anteriorly at the frontoparietal suture.

Once completed, the parietal bones can be gently separated, exposing the lateral cerebral hemispheres. The membranous structures over the sagittal sinus are cut, and the sinus with the falx cerebri is detached posteriorly at the occipital lobes.

An arcuate incision is then made to isolate the occipital squama, reconnecting medially above the foramen magnum. This maneuver also removes the transverse sinuses, torcular Herophili, and posterior cerebellar tentorium, together with the sagittal sinus and falx.

The skull is then immersed in a formalin‐filled container large enough to allow underwater dissection and brain removal. The cranial nerves, medulla, vascular pedicles, and ligamentous attachments anchoring the brain to the skull base are carefully severed. The brain can then be slid into the fixative for preservation and subsequent analysis.

## 4 DISCUSSION AND CONCLUSION

Fetal maceration, which develops after prolonged intrauterine retention in a warm fluid environment (approximately 35°C), leads to accelerated and profound degenerative changes that severely compromise tissue integrity and significantly hinder autopsy procedures. Although such cases were historically regarded as having limited diagnostic yield, the routine exclusion of macerated fetuses from post‐mortem evaluation is now an obsolete and unjustified approach, given the critical clinical, prognostic, and medico‐legal relevance of determining the cause of intrauterine death. When performed according to standardized and methodologically sound protocols, fetal autopsy not only enables the reconstruction of the underlying pathophysiological mechanisms of death, but also provides essential information for genetic counseling, future pregnancy planning, and medico‐legal assessment of cases in which professional liability may be questioned. Failure to conduct a post‐mortem examination substantially limits these opportunities, with potential repercussions for maternal reproductive health, psychological well‐being, and the completeness of the medico‐legal documentation available to judicial authorities and families [9].

In this context, the water‐dissection technique constitutes a practical, cost‐effective, and easily reproducible methodological innovation capable of addressing many challenges associated with advanced fetal maceration.

In cases of suspected fetal hydrops, prolonged retention in the uterus effectively prevents accurate and realistic measurement of the increased connective tissue volumes resulting from imbibition and distension of the coelomic cavities, particularly the abdominal cavity. While the abdominal cavity can only be hypothesized as collapsed due to maceration after initial distension due to peritoneal effusion, the increase in fluid retained by the dermo‐hypodermic tissues can still be documented in cases of non‐excessive retention and before the evolutionary phase toward the papyraceous fetus.

Immersion of the fetus in water induces natural buoyancy, which gently distends the viscera, reduces the adhesion and fusion of anatomical planes, and minimizes structural collapse. This, in turn, facilitates organs identification and markedly enhances structural visibility. As a result, the autopsy can be meaningfully extended to cases that would otherwise be considered only marginally or superficially evaluable, avoiding inconclusive or diagnostically uninformative examinations.

The application of this technique to 17 consecutive cases characterized by prolonged intrauterine fetal retention, with gestational ages between 16 and 36 weeks clearly demonstrated its effectiveness, achieving consistent and reliable separation of the internal organs and preventing the collapse and tissue fusion typically observed in advanced maceration. This was achieved through the buoyancy effect of water, which facilitated gentle expansion and separation of soft tissues, improving visibility and preserving anatomical relationships.

Organs of greater consistency—particularly the lungs, heart, and kidneys—were relatively easy to sample. By contrast, the spleen, adrenal glands, and gastrointestinal tract, although less consistent, still permitted morphologically informative sampling. The liver showed the greatest compromise; in advanced cases, it was nearly liquefied. Nevertheless, overall size and vascular connections could always be evaluated.

A particularly significant finding was the ability to identify and sample structures usually difficult to locate in marked maceration, such as the thymus and gonads. Only through the distension and clear separation of anatomical planes achieved with this technique was precise recognition and isolation possible.

**FIGURE 7:**
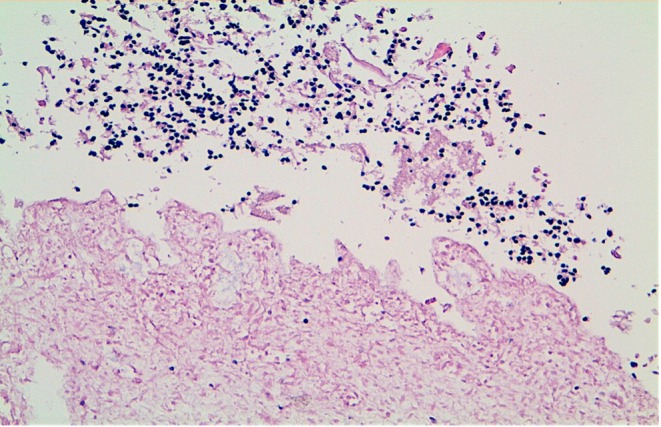
Duodenum section (H&E 10×). Severe and diffuse maceration with preservation of the muscular layer and loss of the mucosal layer.

**FIGURE 8:**
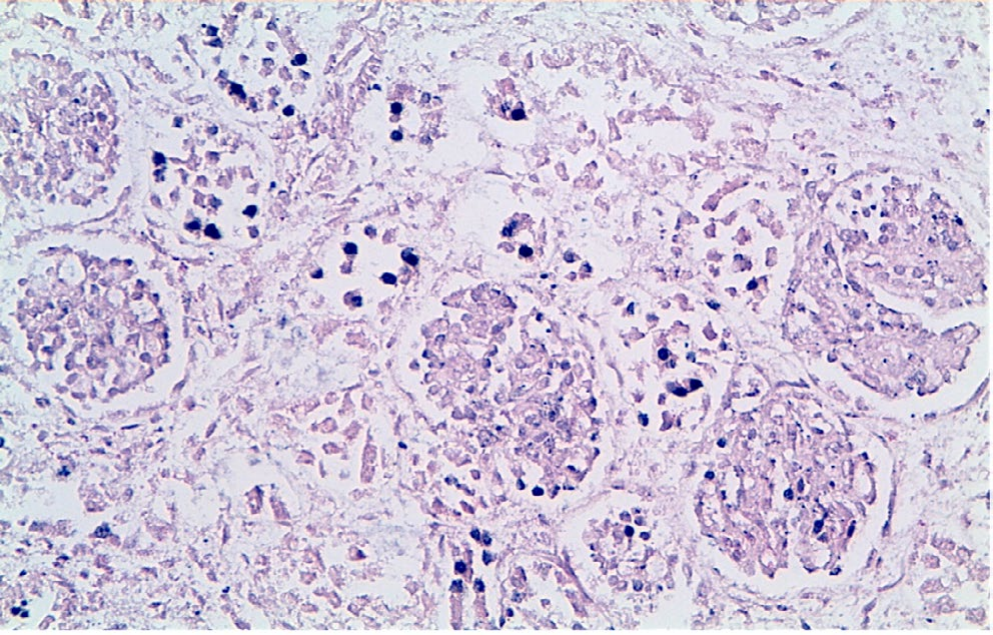
Kidney section (H&E 40×). Diffuse autolysis with preservation of glomeruli and loss of renal tubules.

All organs were subjected to histological examination with hematoxylin–eosin staining, which yielded the following findings; although it did not provide improvements in terms of morphological definition, it nevertheless proved useful for the assessment of macroscopic features:

- Diffuse autolysis, especially in epithelium, while relative preservation of connective tissue (useful for estimating retention duration);
- Advanced maceration of the gastrointestinal tract, with complete loss and partial preservation of the muscularis (Figure 7); and
- Renal autolysis, characterized by the disappearance of the tubules with relative preservation of the glomeruli (Figure 8).

**FIGURE 9:**
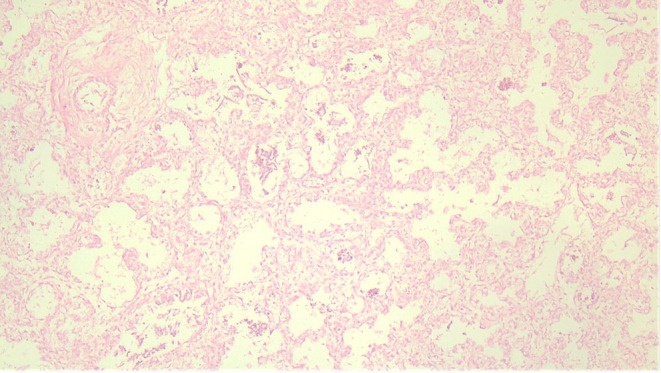
Lung section (H&E 10×). Diffuse autolysis with preservation of sacculate histoarchitecture with microcalcifications.

**FIGURE 10:**
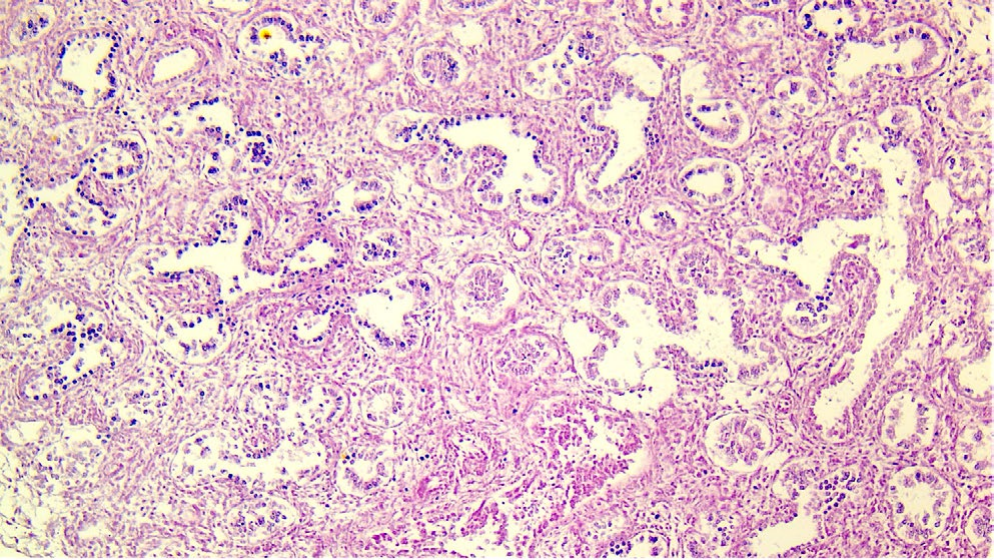
Lung section (H&E 10×). Diffuse autolysis with preservation of canalicular histoarchitecture.

Macroscopic pathological alterations were rarely observed, mainly in vascular structures of the cardiac pedicle and aortic trunk. However, preservation of the organ histoarchitecture was consistently documented, allowing exclusion of major abnormalities and enabling general morphological classification, particularly for developmental staging (Figures 9 and 10) [10].

**FIGURE 11:**
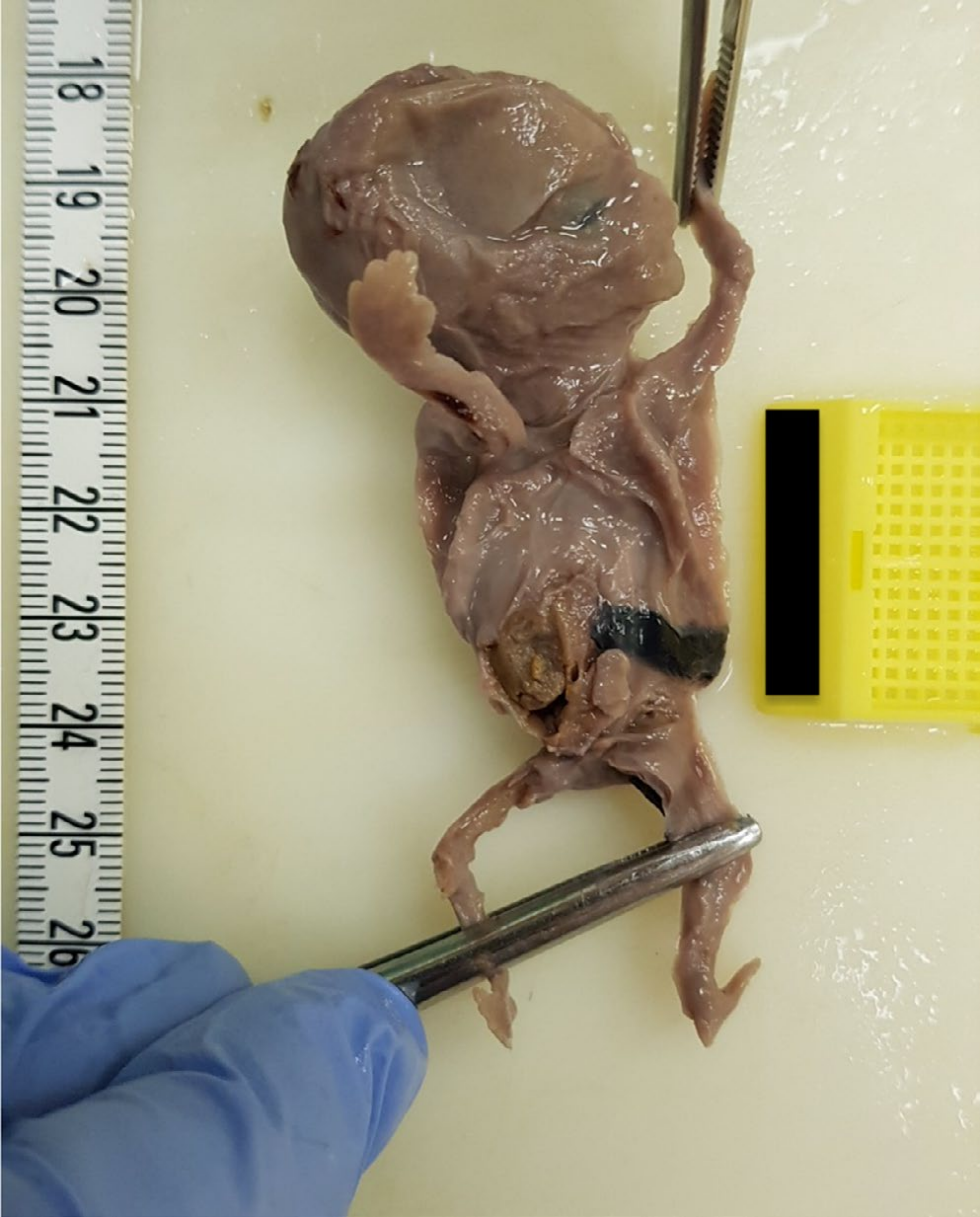
Anterior view of a fetus at the 16th week of gestation, progressing toward fetus papyraceus without the underwater technique.

**FIGURE 12:**
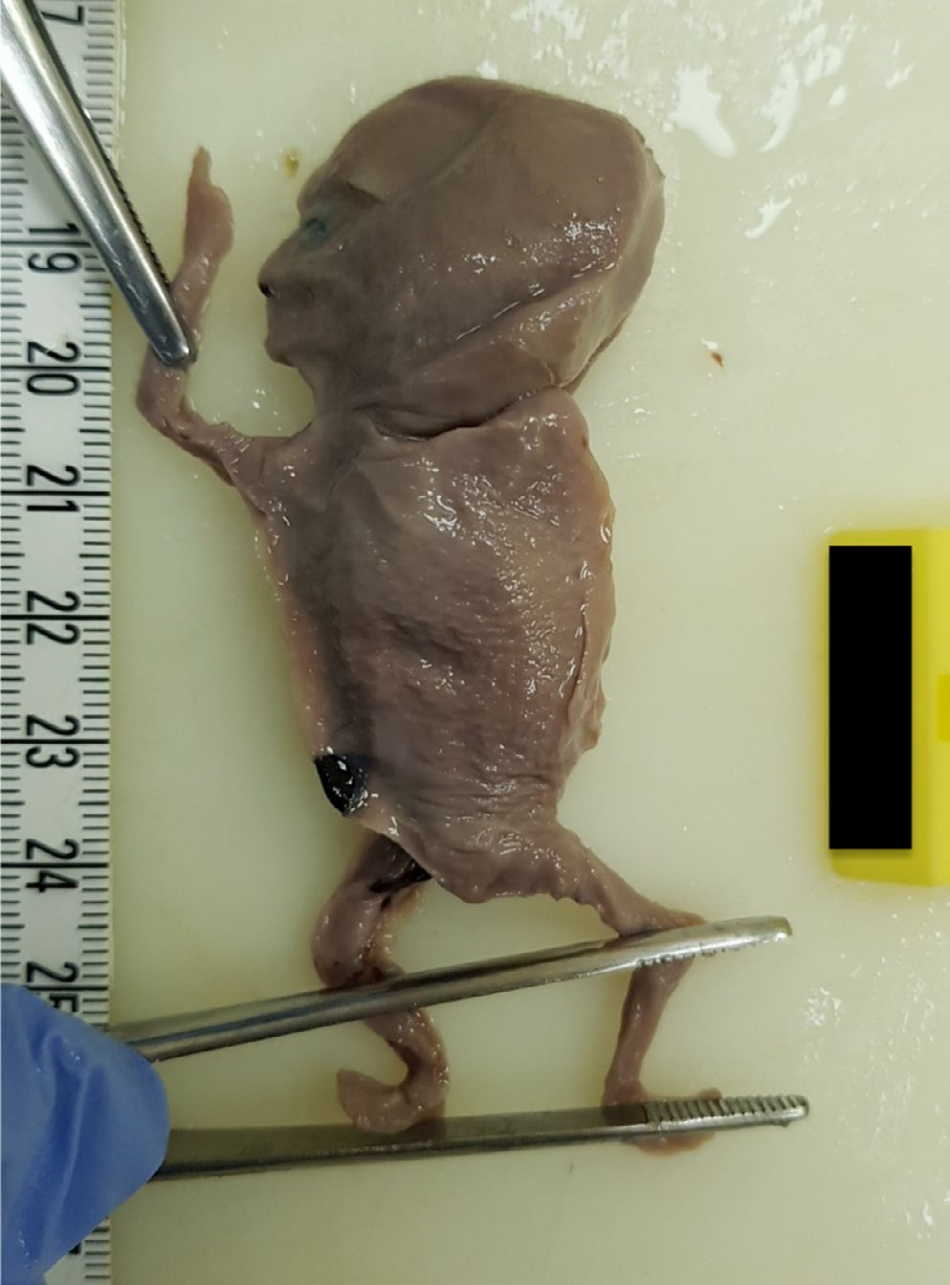
Posterior view of a fetus at the 16th week of gestation, progressing toward fetus papyraceus, without the underwater technique.

For comparative purposes, an age‐matched macerated fetus at 16 weeks' gestation previously examined using conventional (non‐submerged) dissection was retrospectively reviewed (Figures 11 and 12). Gross and histological findings were compared to assess potential immersion‐related artifacts and differences in tissue preservation attributable to the underwater technique.

**FIGURE 13:**
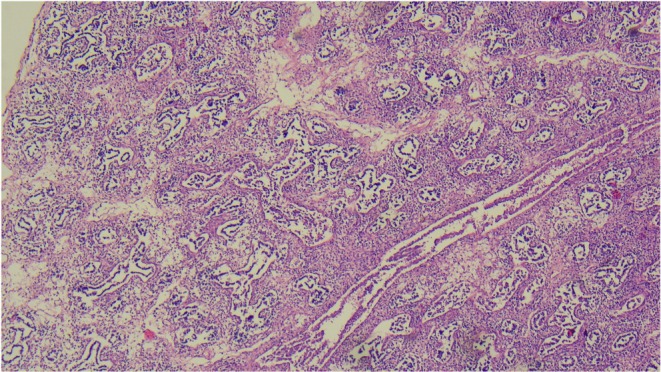
Lung section (H&E 20×).

**FIGURE 14:**
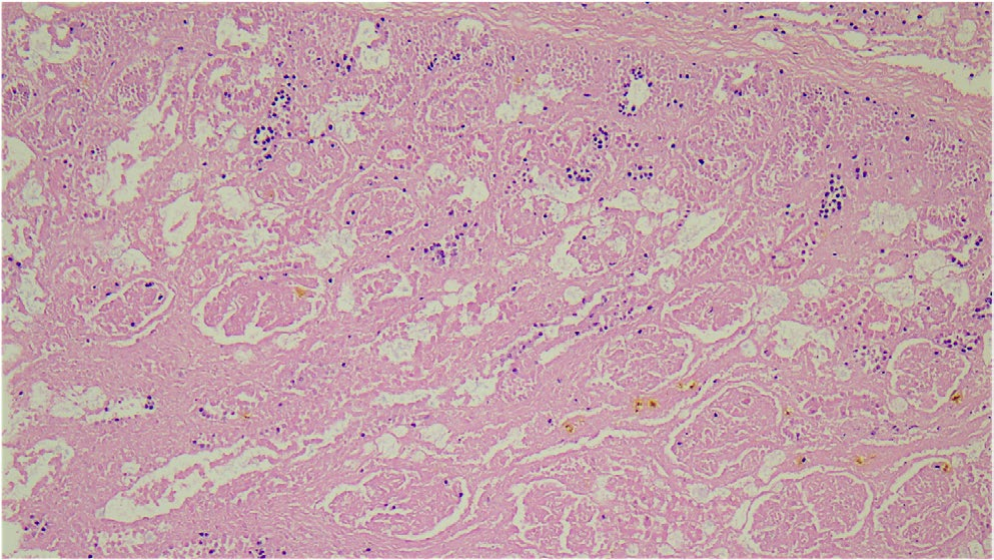
Kidney section (H&E 20×).

The degree and pattern of autolysis were comparable in both cases, and no immersion‐related artifacts—such as tissue edema, artificial separation of layers, or structural distortion—were observed (Figures 13 and 14). These findings suggest that the underwater dissection method does not introduce histological artifacts and does not interfere with subsequent microscopic assessment.

The chronological estimation of intrauterine fetal retention becomes only moderately reliable after approximately one week after death, as gestational age reference charts for fetal growth are constructed on a weekly basis. However, the application of these tools assumes regular growth, a condition often not verifiable in cases of intrauterine death, particularly in the second and third trimesters, when chronic fetal distress may compromise development, potentially leading to progressive intrauterine growth restriction (IUGR) and ultimately culminating in fetal demise.

In this context, fetal autopsy represents an indispensable diagnostic tool, provided that it is performed according to strict procedural standards and includes appropriate collection, fixation, and preservation of anatomical specimens. Histopathological evaluation allows not only for a more accurate estimation of the timing of death but also for the reconstruction of the pathophysiological pathway leading to the event. These aspects are fundamental for medico‐legal assessment, especially when professional liability is suspected or when detailed explanations must be provided to judicial authorities or bereaved families.

A particularly relevant area concerns the investigation of congenital malformations. Systematic examination of all organs enables not only the identification of macroscopic and microscopic abnormalities but also the confirmation of the normal morphology of unaffected systems, supporting the syndromic classification of observed anomalies and facilitating differential diagnoses among isolated, sequential, or syndromic conditions [11].

In conclusion, autopsies performed on fetuses in an advanced state of maceration, when conducted using the water dissection technique, retain substantial diagnostic value. This approach enables documentation of the orthotopic position of the viscera, the anatomical‐topographical relationships, and the overall organ histoarchitecture, even though cytological detail will inevitably be lost. Despite these limitations, the findings obtained are highly satisfactory, permitting the identification of malformations, dislocations, and significant morphogenetic anomalies, thereby confirming the clinical and diagnostic utility of this method.

From a medical‐legal point of view, the adoption of this methodology is highly significant, as it enables the acquisition of objective, well‐documentable, and diagnostically adequate specimens for histological, microbiological, and genetic analyses, even in fetuses that have undergone prolonged maceration. This ensures greater reliability, transparency, and traceability throughout the diagnostic process and reduces the risk of litigation arising from diagnostic omissions or procedures rendered inconclusive by technical limitations.

The systematic inclusion of macerated fetuses in autopsy protocols is not merely a technical advancement, but an ethical and scientifically grounded obligation [9], ensuring appropriate protection for both the affected family and the healthcare professionals involved.

## CONFLICT OF INTEREST STATEMENT

The authors declare that they have no known competing financial or non‐financial interests that could have appeared to influence the work reported in this paper.

## ETHICAL APPROVAL

This study did not involve living human participants or animal experimentation. All procedures were conducted in compliance with relevant ethical standards for post‐mortem examinations and with institutional guidelines. No patient‐identifiable information was collected or published.

## Supporting information


**Figure S1.** Initial section of the skin with double Y incision.

## Data Availability

The datasets generated and/or analyzed during the current study are not publicly available due to privacy and ethical considerations but are available from the corresponding author on reasonable request.
