# Supplementary figures and images for: Advancing fetal autopsy in cases of maceration: Underwater dissection technique and its forensic relevance
Source: J Forensic Sci. 2026 Apr 12;71(4):1821–8. doi: 10.1111/1556-4029.70336 (PMC13340943; doi:10.1111/1556-4029.70336)

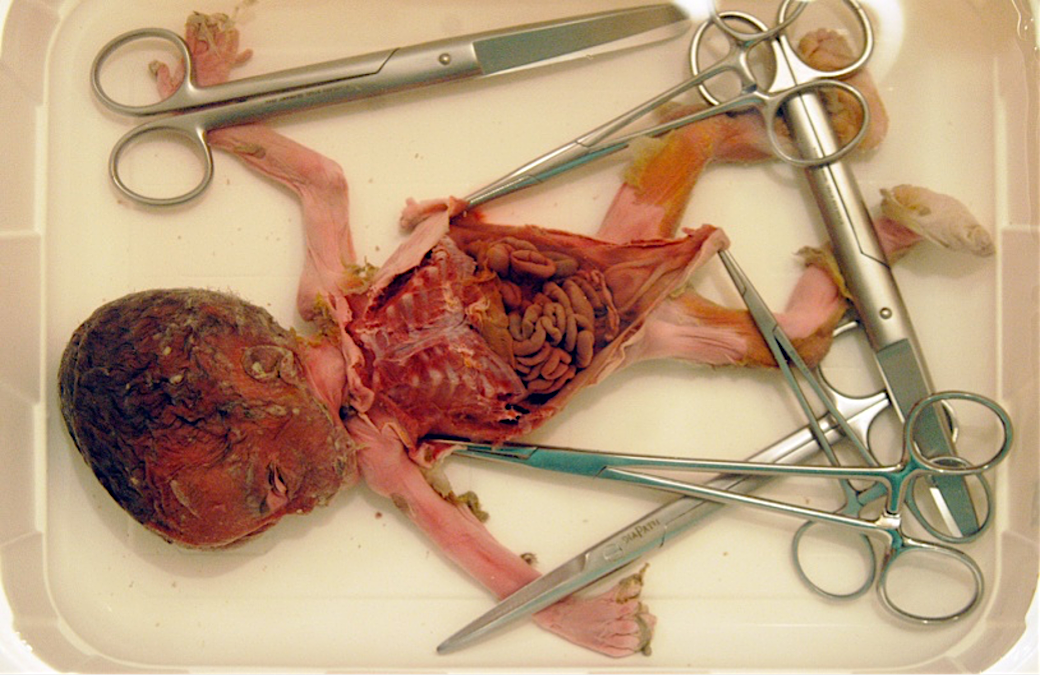

Supplement: Supplementary file 1 — Figure S1. Initial section of the skin with double Y incision. [file JFO-71-1821-s001.png]
